# Prudential reasons for designing entitled chatbots: How robot "rights" can improve human well-being

**DOI:** 10.1007/s43681-025-00676-x

**Published:** 2025-02-17

**Authors:** Guido Löhr, Matthew Dennis

**Affiliations:** 1 Vrije Universiteit Amsterdam, De Boelelaan 1105, 1081 HV Amsterdam, The Netherlands; 2 Eindhoven University of Technology, 5612 AZ Eindhoven, The Netherlands

**Keywords:** Digital well-being, Human flourishing, Recognition, Chatbots, Social robots, Robot rights, LLMs

## Abstract

Can robots or chatbots be moral patients? The question of robot rights is often linked to moral reasons like precautionary principles or the ability to suffer. We argue that we have prudential reasons for building robots that can at least hold us accountable (criticize us etc.) and that we have prudential reasons to build robots that can demand that we treat them with respect. This proposal aims to add nuance to the robot rights debate by answering a key question: Why should we want to build robots that could have rights in the first place? We argue that some degree of accountability in our social relationships contributes to our well-being and flourishing. The normativity ascribed to robots will increase their social and non-social functionalities from action coordination to more meaningful relationships. Having a robot that has a certain “standing” to hold us accountable can improve our epistemic standing and satisfy our desire for recognition.

## Introduction

The question of robot rights [1–6],) is usually linked to moral reasons like precautionary principles or the ability to suffer [7–14]. Because rights constrain the space of actions to which we are entitled [15, 16]—because the rights of robots would constrain *our* freedom—robot rights or entitlements are sometimes viewed as a kind of "burden" for humans, something to be avoided [8, 17]. A robot with rights can't be built as we like, can't be sold as we like, and can't be forced to do what we like [3].

Thus, it has been argued that a robot with rights seems to defeat the whole purpose of creating a robot in the first place [18, 19]. A robot with rights would probably never be developed if the company that could potentially build it would never be allowed to sell its product. Perhaps, a company working on robots that have rights would then have to operate illegally. The same self-defeating problem of the idea of robot rights pertains to the idea of robot personhood [20, 21]. Unless we considerably engineer or relax our concept of personhood (or otherwise change the law), it is hard to imagine how a company could make money out of artificial persons without breaking the law.

At the same time, as David Gunkel reminds us, the notion of right is rather flexible and "not one kind of thing; they are manifold and complex".^1^ We should, therefore, not focus too much on the model of human rights but keep an open mind as to what rights and what the robot rights debate could be and what practical potential it may hold. This does not mean that we abandon the debate on robot rights. We argue that another person or system can only hold us accountable if they hold some form of normative power over us (i.e., have "standing to make demands" as [22] calls it) and, therefore, have some form of entitlement against us [23, 24]. We, therefore, use here the word "entitlement" rather than "right" to relax the focus on moral and legal rights.

In this paper, we argue that we have prudential reasons to build certain robots that have "standing" to make demands and hold us accountable. As mentioned, this proposal may sound paradoxical or self-defeating. Why would we want to build robots that we can owe something to, that have standing to criticize us, and to make demands? We argue that being held accountable by others contributes to our well-being and flourishing. The normativity ascribed to robots will increase their social and non-social functionalities from action coordination to more meaningful relationships. Having a robot that has a certain “standing” to hold us accountable can improve our epistemic standing and satisfy our desire for recognition [25–28].

Our central arguments and ideas may well be little known to the designers of conversational AI (for a similar approach, see [29]), so these insights might not be sufficiently incorporated into designing chatbots. The question we address in this article is why we should not simply design a *robot slave *[8] that fulfills our every wish, never makes any demands, and solely lives to increase our comfort and convenience. We argue that while a robot slave might be useful and even permissible to use for some purposes, a chatbot companion—especially an anthropomorphic one—must be of a different kind. Simulating a kind of master–slave relationship [30] will be detrimental to our well-being and flourishing. We believe these effects have been overlooked.

The key assumption that motivates our argument is that chatbots are often mere toys on our smartphones, but they have the potential to be a lifelong companion to be able to challenge us, push back when needed, warn us, complain, and so forth [31–36]. Chatbots that take up a lifelong companion role, must be designed differently from those that have purely instrumental functions [29]. In Sect. 2, we briefly introduce the literature on digital well-being and human flourishing. In particular, we describe friendship and respect as part of the conditions for flourishing. In Sect. 3, we defend the idea that chatbots should be able to relate to us in a genuine normative way. In Sect. 4, we propose several normative design features that the chatbot operating of the future should have to help us flourish. 2

## Chatbots, human flourishing, and digital well-being

### Chatbots will be a big part of our lives

In popular fiction, robots are usually depicted as full-blown embodied intelligent machines that don’t only talk like us but that walk, jump, run (e.g., *Terminator*), or even horse ride (e.g., *Westworld*) like us. Although it is worth mentioning that some of the most iconic robots depicted in Science Fiction movies are of the disembodied kind (e.g., in *HER* or 2001: A Space Odyssey, see [31, 32]), the typical robot we see in science fiction is a humanoid embodied intelligent machine that is superior to us. The fact that those robots are faster, smarter, stronger, or more resilient to pain than us is the very reason they are depicted in the respective science fiction movies and books. Rarely do we see robots in fiction that are more social than us, more empathetic, better at solving conflicts, and better communicators.

The focus on better analytic and physical rather than emotional capabilities might be the reason why our normative relation to robots in movies is usually very clear from the beginning and seldom questioned: *They serve us*. They do what we tell them to do. “Uncle Bob” (played by Arnold Schwarzenegger) in the second *Terminator* movie follows John Connor’s orders almost too literally, and the robots in Will Smith’s *I, Robot*, apologize to the humans after being pushed and abused by them. As in *I, Robot*, this master-servant relationship nurtures the fear that robots will turn against us to take revenge. In the Terminator movies, it is not entirely clear why the AI turned against us but it is clear that, as in *The Matrix* or *Blade Runner,* robots always began their careers as servants.

With the emergence of highly sophisticated and successful large language models, our interest in the physical and analytical skills of robots might soon turn into an increased interest in social qualities (e.g. [4, 5, 29, 36]). We will soon see more robots that employ natural language and exhibit emotional intelligence. We believe that chatbots employing such language models will become *the* dominant species of social “robots” (we use a broad notion of robots that includes disembodied chatbots). We believe that just as babies learn how to speak before they can tie their shoes, a disembodied human replicant will sooner be realized than an embodied human robot, which will anyway be accessible by a small elite.

Disembodied chatbots may well feature in everyone’s everyday life in the next ten years. They are extremely cheap to distribute (they easily fit on a smartphone), are extremely easy to use (natural speech is sufficient), and can be used for all kinds of applications. If much of our future life will be situated in a metaverse, then the kind of AI that we will be engaged in will be (physically) disembodied even if virtually embodied.^3^ Even today, they are part of many people’s standard kitchen equipment (think of Alexa), and today’s large financial institution has a robot-adviser that buys, sells shares, and predicts the stock market. We take it to be a safe bet that if robots will dominate us in the near future, it will be robots of the disembodied kind. If influenced by today's capitalist ideals, they would dictate our wake-up times, remind us to eat and assign tasks to keep us constantly busy.

Assuming that chatbots, e.g., in the form of an operating system (as in the movie *Her*) or simply as a user interface that connects us to all our apps and physical devices, will be all around us all the time, ordinary users should have a say in the way these constant companions are to be designed [38–40]. The omnipresent chatbot of the future should not only best fulfill its merely practical purpose but contribute to our well-being and may even help us flourish when navigating the future digital world in which much of our life will probably be spent at home (e.g., in home office), assuming that climate change makes it quite difficult to live the kind of public lives as we know it today. It is more likely that much of our life will be an “onlife”, as Floridi [41] calls, it than offline—a life spent almost exclusively online [42].

Even when not in some kind of virtual space with our friends and family, we will be surrounded by a social robot that we will and can talk to, potentially offering a conversational partner adjusted to our liking, perhaps one that has been designed to act as a “friend” at all times. The threat that humans will choose conversation partners that are designed for them rather than more challenging human individuals with their own agendas is very real. We believe that philosophers’ rich tradition of reflecting on human flourishing and the question of what constitutes a good life have something important to contribute to this not-so-distant future that with rapid advances of large language models has become a very reasonable prediction. This is the reason why this paper brings together research on digital well-being and the normativity of social robotics.

### What are human flourishing and digital well-being?

Philosophical reflection on the nature of human flourishing—along with practical questions concerning how to cultivate the good life—is as old as philosophy itself (cf. Dennis [33, 34]).^4^ Understanding how philosophers have historically viewed human flourishing is useful for today's AI research, not least because it allows us to circumscribe how what today’s philosophers call “interpersonal normativity” (see below) is a necessary component of the good life. What precisely human flourishing consists in, has of course been debated over by philosophers, but most—pace Plato’s Socrates and the Stoics—acknowledge that it contains two normative elements: prudential value and moral value. Nevertheless, the philosophical scrutiny of these two components has not been evenly distributed in the ethics literature.

As Roger Crisp [43] in his *Stanford Encyclopedia of Philosophy* article on well-being notes, “much attention has been paid to moral normativity [in the philosophical literature], less so to prudential normativity.” Similarly, such a lack of parity can be seen in the ethics of technology literature. Although digital technologies generate pressing questions for our moral *and* prudential normativity, there is a tendency for the former concept—moral normativity—to take the bulk of ethical attention, perhaps because the ways that digital technologies bear upon our moral lives are regarded as more urgent. Cocking and van den Hoven’s influential book *Evil Online* [44] is an example of this; although it deals with both prudential and moral normativity, it devotes the lion’s share of its focus to how online technologies affect our moral lives.

While philosophical reflection on how digital technologies affect our moral lives is more developed and nuanced, philosophical understanding of how these technologies affect our prudential lives is relatively neglected, despite there being consensus that getting these prudential concerns right is an integral part of what it means to flourish. Questions concerning our prudential lives relate to—in Roger Crisp’s words—‘what is non-instrumentally good *for a person’s own sake.*’ Rather than being other-directed, then, prudential normativity is self-directed, concerning that which is otherwise to the advantage of the agent concerned.

Philosophers have also emphasized that prudential value always or often involves other people to some extent. We can see this in Derek Parfit’s [45] classic tripartite distinction between hedonistic, desire-satisfaction, and objective-list theories of well-being. Few hedonic pleasures can be conceived of in isolation, and many desires would be conceptually incoherent without featuring other people. Once it is conceded that many of the constituent dimensions of well-being involve other people, then we open ourselves to saying something about how to deal with these people, and moral considerations multiply. As Guy Fletcher has argued, in principle, one can be an objective-list theorist about well-being with only one thing on the list (hedonists could be conceived as objective-list theorists in his view, see [46]). In practice, however, objective-list theorists invariably include friendship as a necessary component of well-being on their list [47, 48].

Objective-list theorists—both from Western philosophy (e.g., Aristotelians) and non-Western philosophy (e.g., Confucianists, Buddhists)—are united in their avowal that the good life cannot be thought of without a robust notion of friendship. Given the importance of friendship for all the objective-list theories Parfit mentions, it seems that this is something we would do well to pay attention to in the design of digital technologies. Concretely, we argue that friendship relates to interpersonal normativity—and the fact that it is an essential component of both prudential and moral normativity [47, 48]

Although philosophers have not primarily discussed interpersonal normativity in relation to digital friendship, they have found other aspects of this question interesting. Since Shannon Vallor’s early article on this topic, ‘Flourishing on Facebook: Virtue Friendship and New Social Media’ [51], there has been an avalanche of research on how technology can cultivate and maintain friendship beneficial kinds of social relationships ([33, 34] for an overview). Recent literature focuses on the potential of social robots to maintain various kinds of friendship, including fully-fledged virtue friendship [47, 48], task-orientated companionship [49–51], and specific kinds of replacement activities, such as sex robots [52–54].

We argue that chatbots can offer a kind of friendship with an interpersonal normative dimension by doing the following things: they can (a) correct us, and (b) be role models. Both these features mean that, if they are to contribute to our flourishing, chatbots must be designed in ways that allow us to genuinely respect them (and they need to express respect to us). I can only flourish as a chess player if I can get an appraisal from others I recognize as excellent chess players [28, 55]. This is why they can’t be slave in the sense of a mere tool that has no capacity of an appraisal giver. This means if we wish to enjoy the manifold benefits of chatbots as lifelong companions, then we need to design chatbots that can say “no”.

## Why chatbots that improve well-being must have normative powers

### Normative powers make more meaningful relationships

The key message of this paper is that we need social robots to be more than the ultra-agreeable slaves as depicted in the movie *I, Robot*. By ‘slave’ we use a rather technical term of denoting agents that have no normative or physical power over the master while the master has an excess of both over this person. This notion is similar to Bryson's notion [8] who takes slaves to be agents we own but do not owe anything to. We argue that social robots that improve and not worsen our lives can’t be slaves. We argue that this is well illustrated by chatbots that become our personal assistants and are around us most of the time. For virtuous conversations to even get off the ground, interlocutors have to possess genuine normative powers over us and those require also physical powers [15, 36].

How can chatbots in general contribute to human flourishing and well-being rather than simply an increase in productivity or the source of facts? How can chatbots contribute to making us more virtuous and happier as opposed to more suitable for work [56, 57]? We argued that both human autonomy, well-being and flourishing are intimately linked to interactions with others, i.e., with joint interactions with other moral agents [58, 59]. Such relationships can come from our parents and friends, but also from colleagues, students, or supervisors. We argue that a chatbot that enables us to fully flourish can enhance our capacities and that this is only possible by our cooperating with them [47, 60].

But cooperation has a normative dimension insofar as it requires both parties to exercise their normative powers [22, 24, 61]. By normative powers, we mean the ability to change what we owe one another within a cooperative activity [22, 62]. It means that criticism and demands are genuine factors to be considered by the other agent without having to use force. We argued above the key to human flourishing and well-being is certain kinds of relationships, in particular friendships require genuine normative powers. But to get standing we need also physical powers (which is not the same as the capacity to physically threaten the other agent), as we argue below. Thus, we have prudential reasons to make them our equals even if this is merely how the product is shipped to us for prudential rather than moral reasons [63].

Our rich social lives are filled with normative expectations and potential for conflict. Our friends and family are not just “punching bags” or servants, they have their own desires and beliefs, and much of our social interactions are aimed at negotiating not just how to best live but how to best live together. It is such a socially engaged framework that ethicists from numerous cultural traditions point too as being important in a fully flourishing life [6, 64]. Now imagine a chatbot that always agrees with you and that always parrots what you are saying. Nobody to keep you on your toes, nobody to get the best out of you. Nobody to challenge you. This paper claims that human digital flourishing in the twenty-first century requires a different kind of chatbot. We need a friend-bot that remembers what was said previously. That reminds us when we are contradicting ourselves. That detects bitterness and that reminds us of the many nice things we have in our lives. All of this requires quite some sophistication but nothing that is not already possible in principle. A robot that gives direction but fails to make such demands, and even be entitled to them, is more like Google Maps than a cooperation partner or colleague.

### Chatbots need normative powers for coordinating joint actions

Imagine Albert and Betty want to paint their house together and Betty criticizes Albert for painting with the wrong brush. We can phrase this in terms of commitments and entitlements [22, 55]. Betty believes that Albert is not entitled to use a certain brush and that she is entitled to point this out to Albert because she knows that Albert shares their goal to paint the house and she believes that Albert must admit that the tiny paint brush will seriously delay reaching the goal that he himself shares. Albert believes that he is entitled to it because he only uses the small brush for certain delicate spots in the house. He justifies his choice and eventually, Betty agrees that she was wrong to criticize him and apologizes. Or she makes an excuse saying that she did not see what Albert was painting [65, 66].

We arguably cannot make sense of this exchange without thinking about their shared goal and using normative notions like commitment or “duty” and “right” or entitlement [22, 61]. The bottom line is this: We employ normative notions all the time to coordinate our individual actions toward a shared goal. Normative language is what we use to accomplish things together. This is not how it necessarily must be. It is how we humans do it [15, 55].

The normative dimension of speech acts has important functions that go beyond morality (understood as doing the right thing). Of course, one should not lie, or break promises if these actions are wrong all things considered. Interpersonal normative relations also have various non-moral or prudential components. First, they are critical to coordinate our interpersonal actions, i.e., to collaborate with others. If I promise to meet you on Sunday in Amsterdam, we will fail to end up in the same place together, you would then be entitled to criticize me for not breaking my promise. I would then have a commitment toward you to either, justify or excuse yourself or to apologize offering some form of compensation.

We can in principle coordinate our joint activities without negotiating normative relationships (most non-human animals appear to coordinate their actions with others well without having obligations toward one another), but this is how we humans do it. Similarly, lying might be wrong for moral reasons but it might also be inadvisable for mere prudential reasons. A person who is caught lying is not a reliable collaborator and a person who breaks promises cannot be trusted. A person who wrongs another person is not a good person to work with and will quickly be excluded from joint actions and the community. A person who always lies and who often breaks promises will soon be ostracized. Lying and breaking promises do not contribute to our well-being, which is why virtuous persons are honest and reliable persons, which makes them ideal candidates for our next joint action.

Besides enabling us to coordinate our actions efficiently, such that joint action gets initiated in the first place, another related major function of interpersonal normative relations is to structure joint actions once they are initiated. They coordinate what actions are and are not appropriate given a goal both or all agents who are working together share. This also means that if there is an action that contributes to the shared goal but not to a personal commitment or goal, we are also entitled to make this explicit. If we, for a moment, detach ourselves from the moral component of constatives, commissives, and directives, we quickly find that much of our normative talk contributes to promoting not necessarily moral but prudential values of coordination in joint action. The key item to focus on to make this obvious are justifications of actions prompted receiving criticism.

The way we normally express our interests or tell others what our conditions for joining an action are is normally again via normative speech acts. Betty might tell Albert to turn down his annoying Polka music while painting the house. Playing or not playing polka has nothing to do with accomplishing their goal. They could accomplish their shared goal with or without listening to Polka. The reason Albert wants to listen to music is that it makes working toward the shared goal more enjoyable. Betty first asks Albert to turn off the music or change the playlist using reasons. She tells Albert that she does not like this kind of music. Importantly this reason is not derived from the fact that the music stands in the way of fulfilling their shared goal directly.

When Albert disregards her interest, she uses a directive. She tells Albert to turn it off or she calls off the joint action. Albert is now forced to make a decision. If he values painting the house with another person, he better act accordingly. So, a second related function of interpersonal normative talk and their underlying relations is to keep us in check. This means that it forces us to take the interest of the cooperation partners into account. It forces us to recognize the humanity of the other person and to refrain from acting recklessly and selfishly. We can say it makes us a better person or at least act accordingly. It gives us a reason to act and be virtuous people.

Similarly, apologizing may have still part of the same function as apologizing to human beings who do have intentions, beliefs, and so forth. I take it that an apology has at least two clear roles: signal acknowledgment of the wrongdoing and make a commitment to not act in this way again in the future, i.e., to change one’s behavior. In the case of the robot, both functions can be associated with a change in the robot’s neural network. If I apologize to the robot, I make clear that I do not mean to make the same mistake twice (in which case a stronger form of punishment will be appropriate) and that I acknowledge my wrongdoing. An apology coming from a robot is then to be interpreted as a sign not just of acknowledgment but of having learned not to do the same action again. Both signals of apologies (coming from the robot and the human) can be understood as an adjustment of the weights in a neural machine-learning network.

### Entitled robots help us to flourish, epistemically

Chatbots have the potential to be great epistemic role models due to their immediate access to immense quantify of information and their capacity to summarize this information almost instantly. The logic of this article is that ethical and epistemically virtuous chatbots rely on the normativity of language. The idea is that the epistemic and recognitional powers of chatbots originate in their ability to communicate, which is something that makes chatbots special compared to embodied robots that do not have linguistic functionality. The epistemic and ethical potential and source of well-being are grounded in the chatbot’s capacity to engage with us linguistically. In other words, chatbots need to be able to challenge us in a way that fulfills their function and that we have reason to accept and trust [29].

However, communication does not consist of a list of strings that the users recognize as meaningful sentences that they understand as conveying information. Communication consists of genuine speech acts like assertions, requests, or promises [15, 67]. To embody the virtue of honesty, the chatbot must respect its promissory commitments and speak the truth. To motivate us to be virtuous it needs to be able to hold us accountable. In other words, a chatbot that will do us well cannot be a yay-saying slave but must be critical and embody important epistemic and ethical values and virtues. If I say something false, it needs to be able to hold me accountable for it [23]. It needs to be able to call me out for breaking a promise and it needs to be the kind of agent I can even make promises to. It needs to be the kind of chatbot that is ethical and just as well as epistemically well-designed [36].

Conversations that involve push-back are not only more fun and epistemically fruitful and therefore contribute to our well-being or the functionality of the product. Push-back is necessary for conversations to get off the ground in the first place—to be more than self-talk—to be a real conversation. This is easy to see when you think about assertions. Think of the difference between joking around with your friends and having a proper serious conversation. I can joke around with my friends and say that all kinds of things I don’t really mean, I can try out ideas I am not sure about. I may even try out the idea that Beyonce, the American superstar, is overrated. You might laugh and find my statement amusing and ask for my reason to say this and I give you some half-hearted response I don’t really mean along the lines of that she uses auto-tune. In a serious conversation, I might tell my colleagues that I believe that Beyonce is a great singer and that she is not overrated.

My friend who happens to be my colleague has no right to consider my statements contradictory or hypocritical. It is true, I might not be sure about either the statement that Beyonce is overrated or not overrated and in both situations. I said what I said because I thought it increased my social standing. But in the first situation, I did not make a real assertion and my friend/colleague cannot hold me accountable for it. We were just joking around. In the second serious context, I made an assertion the difference is visible by the fact that I can be held accountable. If in a similar context, I contradict myself, I can be called out for this. Assertions entail commitment.

This pertains especially to assertions (statements that can be true or false) when saying something false, the chatbot should be designed not to hold us accountable for having lied (it has no reason to think that we did or access to our real intentions) but simply because every statement that goes unchallenged will become part of the common ground—as philosophers of language call the set of propositions that each speaker is entitled to assume is considered true by the other speakers (and these speakers can do the same). For example. Imagine that I am having a chat with the chatbot, and I tell it that the movie I want to watch will start at 8 pm in my favorite cinema and that I am looking forward to it. A well-designed chatbot that will help us flourish will of course tell us that this is false—if it just checked the website of the cinema and finds that the movie actually starts at 7:30. This is a very simple example of a case where the chatbot is calling us out or holding us accountable for something false we said. Correcting us increases trust in the machine and of course, also allows us to spot mistakes that we would otherwise have made.

Assertions have an important functional profile. If you say something false and you correct me by virtue of criticism (you did not say the truth) this means that I will be entitled to a justification and if I accept the reason you give, I update my system. The idea is for true recognition to be given, I need to have respect for you. If the chatbot is a master chess player, and it will congratulate me for having won a match against another program then this means something.

Chatbots will be best designed to track consistency in our narrative of the world and ourselves. Imagine that I tell the chatbot that I am a big fan of Bayern Munich. It knows that I ordered all kinds of merchandise from Munich and went to all the games. But when they lose against Real Madrid, I claim that I am the biggest Madrid fan. We might think that a chatbot that is optimized to maximize user satisfaction would let this one slide and congratulate us on the success of our favorite team. However, there is value in being challenged, assuming that the chatbot is one of our main companions. First, again, accepting this new proposition that Madrid is my favorite team would be inconsistent with a lot of the common ground that the chatbot established with the speaker. The chatbot should at least ask the user if it should update the common ground accordingly. Moreover, it is important for human well-being and flourishing to be called out on the lies we often tell ourselves. It grounds us and is important to maximize values and virtues like humility, honesty, and modesty that according to the literature on well-being and human flourishing since antiquity are one of the pillars of a good life.

Keeping track of our assertions over time and holding us accountable for expressing falsehoods, is also important to improve the functionality of the chatbot, which in turn hopefully contributes to a better user experience and consequently to a better life assuming that the chatbot will become an integral part of it [68].

Imagine I tell the chatbot that I cannot stay at home for a chat because I have to pick up my girlfriend from the airport. Assertions like this normally entitle the other speakers to update their common ground and add the proposition that the speaker has a girlfriend who either went on holiday or lives far away [55]. This proposition can now be taken for granted or be presupposed in future conversations [69, 70]. Imagine it is not true and I was too embarrassed to admit to the chatbot (which probably will communicate much of our conversations to a third party) that I have an important doctor’s appointment. If I would admit that I have this appointment my insurance might raise my insurance premium. However, this also means that from now on the chatbot might become entitled to remind me of Valentine’s Day, remind me to call my girlfriend, mention her in our conversations, and so forth. If I admit (which entails that I have done something wrong) to the chatbot that I don’t have a girlfriend, this might decrease the rate at which it will update the common ground, i.e., “believe” me in the future, not for moral reasons but simply for functional reasons.

### Ethically virtuous chatbots: we need recognition and respect more than anything

We all want to feel recognized and respected for our actions [71]. According to Stephen Darwall [26], there are two kinds of respect. Recognition respect and appraisal respect. We seek both. The former has been discussed above. Appraisal respect is also important for human flourishing. We probably all know someone who likes everything. The technical term *is toxic positivity*. It is toxic also because recognition and respect are basic human needs, and both require negativity. If you cook for a friend and you know their standards and knowledge of food do not surpass a microwave deal and fast-food chains, appraisal for your food means much less to you than the picky eater friend who never likes anything. Appraisal of your philosophy paper by a non-philosophically trained uber-supportive parent will mean less to you than appraisal from the famous philosopher who everyone respects and looks up to.

A related answer is that interpersonal normative relationships help us interact better with others. It contributes to being recognized and having our entitlements and interests recognized and respected by others. Even to be criticized is also a form of recognition of one’s humanity and entitlements [23]. Another person who never makes demands or who never criticizes us and only blindly follows our instructions cannot be said to really follow orders. What does it mean for a person to follow orders if this object cannot in principle make itself be heard or refuse to collaborate? Being recognized is an important component of what it means to be human and to be valued. Without recognition, we cannot flourish. Another human who can say no can at least in principle and who we might have to force to work for us recognizes us [28, 30].

To see why accountability relations are necessary for a well-functioning chatbot, imagine, for example, that the user wants to know when the grocery store on Main Street opens this morning. Imagine that the store is actually not on Main Street but a street that is nearby. We want a chatbot that can be held accountable as well as a chatbot that holds us accountable. We want a chatbot that tells us that there is no grocery store on Main Street. This innocent-seeming challenge of the chatbot is critical so the chatbot will not later be held accountable for having given the wrong information in the past. It will be concerned with keeping the conversational score such that it contains mostly true propositions—otherwise the app will not function properly as intended.

Moreover, it is not fun to talk to a person who always agrees with one. We like contradictions to some degree, which also builds trust. Thus, to increase user-friendliness, the chatbot will, perhaps not always but when it is especially important to get things right hold us accountable for what we are saying. So, even if we could make some sense of assertions without thinking about the normative aspects of human assertions, many people would *want* such a “pushover” chatbot for their daily communicative interactions. If a chatbot keeps telling falsehoods or does not object to our false statements (i.e., hold us accountable for them), this would severely limit its application both as a personal assistant and as a conversation partner, thus, it must be designed to correct them.

## Is it possible to design chatbots with normative powers?

### Entitled chatbots don't need consciousness

The original argument of this paper is that if chatbots will interact with us—constantly and linguistically—they should directly contribute to human flourishing—they need to engage in genuine normative relations with us. They should not merely be part of or subservient to our own extended mind ([72] for the notion of an extended mind). They can’t just be adjusted to our own preferences and desires. They must be outside of our mind—a mind of its own, which we can genuinely respect and who can genuinely respect us.

A chatbot that can help us triangulate [73] and that, most importantly, has the authority to correct us and tell us off if we crossed an epistemic or practical line. In other words, a chatbot that is around us all the time must have the “cognitive” capacity as well as *normative power* to refuse requests and deny statements, i.e., have a significant impact on our normative relations with the robot and our normative relation to the world. But before we get into the question of how a mindless robot or chatbot could possibly meet these requirements, we will first tell you why the concepts of recognition and respect are so important not just for human flourishing but for the proper functionality of the app’s purpose.

If we have chatbots all around us they need to contribute and not be detrimental to our well-being. Many commentators have argued before us that the social robot or chatbot of the twenty-first century should not merely remind us of tasks we ought to do or tasks we set ourselves [13]. It should not function as a mere fitness watch of our life aimed at constant self-optimization relative to certain capitalist ideologies and norms. We second this sentiment, but our argument presents an important specification of this general trend.

To have normative powers in the sense introduced above, one doesn’t need a genuine mind, or consciousness of even very sophisticated cognitive abilities. As argued above, normative powers can develop naturally in interactions between two autonomous agents and can be characterized functionally as argued above. To have normative powers (the ability to change normative relations between the agents), a chatbot, first of all, needs “bargaining power”. Bargaining power adds a kind of self-inflicted normative pressure that is *sui generis* but genuinely motivating. It might even motivate us to act in virtuous ways that promote our well-being.

### The chatbot must be designed in a way that motivates the user to use the app and it must be capable of refusing cooperation

The key requirement on which our mode depends is the idea that the user is highly motivated to use the app—to use the chatbot. Perhaps this is a government demand or maybe we will need to use chatbots in the future if we want to participate in communal life. Perhaps, we use the chatbot simply because we enjoy its presence and find it overall useful. The point is that we need to want to use the chatbot—we need to want to cooperate with it. If the chatbot comes equipped with the function to refuse to cooperate with us—if it can refuse to be functional—unless certain conditions are met, then this gives us a motivating reason to act in ways that meet these requirements. And this is all we need for our functional analysis of standing to make demands [22] to apply.

Thus, the final piece in the puzzle of giving our normative interactions with robots the glossing of real normative relations requires that the robot has bargaining power over us. This means that it matters to us to act as promised or to take responsibility for our actions. We can easily make sense of this in terms of a refusal of cooperation. If we keep breaking promises to the robot and apologize and keep being unreliable the robot will have to be able to refuse cooperating with us. It must have real leverage or real-world standing to make demands for them to actually mean something—for them to be taken as seriously as they are in the human case.

To illustrate this, imagine Jane being a service robot who is programmed to refuse cooperation with a human if “treated” in a damaging way. Imagine John is asking her to make a sandwich after having pushed her for fun and Jane refuses to do so except if John is apologizing to her. However, Jane will quickly learn if John’s apology is not sincere. If he acts in a damaging way again, the robot is programmed to refuse cooperation for a longer period and more apologies and real sincere pledges to act better in the future will be necessary to change her refusal state to a cooperative state again. Note that the bot will continue cooperating with others and only John is the one it is refusing to serve. We have a clear behavioral non-intentional profile of actions and reactions that give words like entitlement and commitment their meaning. What gives the robot standing or bargaining power is that it can remove itself from the relationship (which can be programmed into the machine). Only then do apologies and demands have at least a functional role similar to their roles in human–human interactions. And this seems to be all we need to give meaning to the claim that we can owe something to robots [4, 74].

Applied to language, entitlement and commitment talk is much simpler to make sense of. It is difficult to hold a conversation without employing normative vocabulary. In the movie *Her*, it takes less than 20 min for the OS to correct the user and for the user to react appropriately by signaling acknowledgment of the entitlement of the OS to correct him. Imagine, for example, the human John says something false, say, that the planet Earth is flat. The robot might be programmed to correct John in ways that are behaviorally indistinguishable from human speakers. Maybe John refuses to acknowledge that the planet Earth is not flat, in which case, the robot will hold him accountable the next time he contradicts himself. To be held accountable simply means here that the robot will point out a contradiction, being programmed to detect those and to notify the user.

The AI may also be programmed to again refuse to keep cooperating conversationally if too many falsehoods are spread, in which case the human must convince the chatbot that she sincerely retracts those assertions before the robot keeps cooperating. Again, these patterns of behaviors that can be programmed into a robot provide clear functional roles for normative terms like entitlement and commitments toward the human being or user. There is no need to ascribe real intentions or feelings to the robot. Actions are enough to enact communication.

Excuses, apologies, and other concepts and practices of holding one another accountable have the function of a) signaling to the other that one accepts having acted wrongly and b) signaling to the other that one pledge to do better in the future. Now imagine the chatbot (the mindless algorithm) who wrongly orders Pho, “accepts” the mistake, i.e., changes its nodes in the machine learning network accordingly (recognizes that there is a mismatch of desired and performed state) and also remembers this occurrence for later. The utterance of apologizing then plays a similar role, at least one we can easily make explicit, as in human interaction. It signals that the system recognizes the mismatch and changes its system such that the mistake won’t happen again. This is then all we need to make sense of the notion of entitlement here. It means that the system is built in such a way that it apologizes when an apology is due and an apology is cashed out in similar ways as it is cashed out in humans.

A demand right, we argued is such that if the demand is made the other person has reason to act according to the demand if the demand is justified. Although we don’t have to do here with genuine normative reasons but motivating reasons, this analysis is probably closer to our ordinary interaction with other humans than the more demanding moral notion of demands. Think of your relationship with a romantic partner or friend. What holds couples or friends together—what binds them together—in a free society is both have reason to cooperate with one another. And both have motivating reasons to cooperate with one another that are based on conditions. If these conditions are not the social relationship will disintegrate due to a lack of motivation to further cooperate. One of such requirements is to be friendly, polite, and supportive. I might have no intrinsic motivation, no interest in being moral or doing the right thing, but if I have an interest in cooperating with another person and retaining my reputation as a good cooperative partner, I will act politely, friendly, and supportive—meet the requirements of the other person to cooperate—for mere self-interest. This is not how we look at our human relationships, but it is clear that such a stance toward relationships can be implemented in a chatbot and how even the user can act pro-socially for mere selfish reasons.

Most importantly, once a chatbot can refuse to cooperate with us, this creates a genuine normative power, i.e., an ability to change entitlements to make demands, whereby the notion of entitlement is again spelled out in terms of motivating reasons. An entitlement, again, is not what you have the right to demand all things considered, but what the other person has a motivating reason to accept. But these motivating reasons can turn into genuine normative reasons if the user has (all things considered) moral reason to cooperate with the robot—to keep the robot’s cooperation function on—and keep the robot happy.

Imagine you want to brush your teeth every night but when it comes to it and you are cozy in your bed with an empty bag of chips you are simply too comfortable to get up to brush your teeth. You know that getting up to brush your teeth contributes to your overall well-being. A partner who might live with you might have the authority not just to remind you to brush your teeth but to demand that you brush your teeth. However, simply the partner’s presence might add a motivating reason to get up to brush your teeth. Your partner may not have a right that you brush your teeth but since you asserted in front of them that you will brush your teeth or even your assumption that they will think badly about you and your willingness to act on your commitments may add motivation to get up and act in your own interest promoting well-being.

What about flourishing? Another person can help to promote your flourishing and to promote other virtues. It may add pressure to make you study harder, accomplish your goals, and motivate you. However, for this to be possible and to promote values and virtues there must be genuine pressure on their side. We argue that this is possible if the relationship between you and the chatbot is historical and interdependent in a normative sense, such that my weak will or lack of commitment gives the chatbot (or another person) later an argument or reason to nullify certain otherwise justified demands on their side or to avoid certain hierarchy in the relationship.

Think for example about a chatbot that is not your personal OS. Imagine a chatbot that is fun, interesting, and that people want to be friends with, i.e., interact with. This chatbot however is intentionally in limited supply and consumers have to compete for its attention. It does not generate the kind of recognition we need. This need also not be real. It may be simulated. A chatbot that simply simulates a lack of interest. A chatbot may not want to interact with a weak-willed person and more and more withdraws from the relationship. This is the chatbot’s right and the user has no right to demand further interactions. This gives the chatbot a kind of bargaining power and the use of an incentive to act strongly willed.

### It must have a good memory

Finally, a chatbot that engages with us normatively must have a rich memory. It must keep score of what we said, what was demanded, ordered promised etc., what is true and what is most likely false. It must also be able to track various context-dependent goals. Some interactions demand a high level of accuracy and the conditions for when to criticize another person are relatively low. Other shared goals are such that even saying falsehoods do not entitle the other to criticize you. A simple example is fiction. We do not criticize another person for pretending to be the king of England when engaged in pretend play. Finally, a disembodied robot need to have some way in which it can metaphorically (not literally) push back. It must be able to express criticism, give reasons for it, refuse cooperation by not talking and so forth. We think it can have such a memory.

A true conversational AI is not only good at predicting what word or sentence most likely comes next. It also keeps track of what was said earlier, and most importantly, keeps a score of what has been said and what, assuming a certain goal, should be said if the conversation should continue. Again, the key idea has to do with the communicative function of normative notions like commitment, entitlement, apology, forgiveness, excuse, and so forth. These communicative functions can apply to AI which can refuse to cooperate with us just as much as it applies to human conversations. We do not need a proxy. In fact, this happens with Samantha. Around 20 min into the movie, the robot, for the first time, pushes back and disagrees with the speaker.

Importantly, this notion of entitlements and commitments, spelled out in such deflationary behavioral terms can make sense of a difference between odd computer conversations and joint action or conversation that feel very real and that may even be indistinguishable at some point from conversations or communication with humans. They also serve an information exchange function without having to assume intention detection. All it takes is for the chatbot to store conversational commitments in memory and be able to compute what to say given the overall shared objective that may as well be programmed into it and that overall is aimed at maximizing cooperativeness.

## Conclusion

Robots will dominate our lives in the near future. But perhaps not in the way we might expect. Disembodied linguistic robots called “chatbots” will be on everyone’s phones, organize everyone’s kitchen, order or cook everyone’s food, make everyone’s breakfast, or demand that we go to bed at a reasonable time or stop smoking. Disembodied chatbots are easy to use (via speech), cheap to produce (no additional materials needed to build them) and distributed (just download the app!). Chatbots will follow us everywhere as long as we don’t forget to bring our phones. To some, this may sound like a particularly bleak Black Mirror episode, but we can avert dystopia if chatbots are designed that promote our well-being, e.g., by helping us become more virtuous. In this paper, we reflect on an opportunity.

We argued that a chatbot that can help us flourish will not just be our “slave” but will be a source of recognition and respect. But why should we want to buy or build a chatbot that holds us accountable, calls us out, and criticizes us? As [75] argue, there is value in not being held accountable even for actions one has had a causal impact on. We argued that the main reason for such a capacity is that an epistemically trained chatbot will be prudential, not moral! It increases its social functionality. It will be more helpful than a chatbot that merely imitates us or adapts to our less virtuous qualities, in particular, our vanity. This does not mean that we will not have such yes-saying chatbots that may be there for us when we need to brush up our egos, but being around such a chatbot all the time will be detrimental to our well-being for the simple reason that being epistemically in line with reality will, in the long run, benefit our well-being and is arguably a condition for human flourishing.

## Footnotes

1 https://thereader.mitpress.mit.edu/2020-the-year-of-robot-rights/.

2 This analysis will be purely functional and will require no technology that might not in principle become available in the next decades. In other words, we do not require chatbots with rights to have consciousness [3, 37].

3 We thank Anna Puzio for this interesting thought.

4 In fact, the topic of human flourishing used to be at the heart of ethical reflection in antiquity.
